# Optimizing pediatric vesicoureteral reflux management: a single-center experience with contrast-enhanced ultrasound in reducing radiation exposure and antibiotic use

**DOI:** 10.1186/s13052-025-02164-8

**Published:** 2026-02-19

**Authors:** Sonia Tamasi, Anna Masucci, Francesca Antonia De Chiara, Alessandra Chiera, Maria Tardi, Luigi Martemucci, Massimo Zeccolini, Rosamunda D’Arcangelo

**Affiliations:** 1 Department of General Diagnostic Imaging, A.O.R.N. Santobono Pausilipon, Naples, Italy; 2 Department of Radiology, University of Naples Federico II, Naples, Italy; 3 Department of Radiology, University of Molise, Campobasso, Italy; 4 Faculty of Medicine and Surgery, University of Firenze, Firenze, Italy; 5 Department of General Pediatrics and Pediatric Immuno-Rheumatology, A.O.R.N. Santobono Pausilipon, Naples, Italy; 6 Department of Kidney Transplantation, Pediatric Nephrology and Dialysis, A.O.R.N. Santobono Pausilipon, Naples, Italy

**Keywords:** Vesicoureteral reflux, CEUS, Pediatric nephrology, Antibiotic therapy

## Abstract

**Background:**

Vesicoureteral reflux is the most common pediatric urological anomaly and a leading cause of urinary tract infections in children. Traditional follow-up typically relies on voiding cystourethrography, which exposes young patients to ionizing radiation. The introduction of contrast-enhanced ultrasound provides a radiation-free alternative with comparable diagnostic accuracy. This study aims to demonstrate that the use of contrast-enhanced ultrasound can shorten follow-up intervals, thereby reducing the duration of antibiotic prophylaxis.

**Methods:**

A prospective observational study was conducted between 2019 and 2024, involving 1050 children with urinary tract infections, hydronephrosis, or a known history of vesicoureteral reflux. The cohort included both male and female children, aged between 1 and 15 years. Diagnosis was confirmed using conventional ultrasound and contrast-enhanced ultrasound. Patients with grade III to V vesicoureteral reflux underwent contrast-enhanced voiding urosonography at 12 months, replacing the standard voiding cystourethrography typically performed at 24 months. Clinical data were collected on reflux severity, gender distribution, and changes in antibiotic use.

**Results:**

Early reassessment using contrast-enhanced voiding urosonography resulted in a significant reduction in the duration of antibiotic prophylaxis. A substantial number of patients showed resolution or downgrading of vesicoureteral reflux at 12 months, allowing for earlier discontinuation of treatment. The diagnostic accuracy of the ultrasound-based approach was consistent across age groups and genders.

**Conclusions:**

In our single-center experience, the incorporation of contrast-enhanced voiding urosonography into follow-up protocols for pediatric vesicoureteral reflux has been demonstrated to be a safe and effective alternative to traditional imaging. By allowing earlier reassessment without radiation exposure, contrast-enhanced voiding urosonography facilitates the safe discontinuation of prophylactic antibiotics, thereby enhancing patient safety and contributing to the reduction of antibiotic resistance.

**Trial registration:**

Not applicable. This is an observational study and does not meet the criteria for mandatory trial registration.

## Introduction

Vesicoureteral reflux (VUR) is the most common urological anomaly in pediatric patients and is closely associated with urinary tract infections (UTIs). The condition is characterized by the retrograde flow of urine from the bladder into the ureters or kidneys, posing a risk of kidney damage, hypertension, and chronic kidney disease if not properly managed [1]. Early diagnosis and continuous monitoring are essential to prevent long-term complications, especially in cases of high-grade VUR. Traditionally, the diagnosis and follow-up of VUR have relied on voiding cystourethrography (VCUG), which, while effective, exposes patients to ionizing radiation, a particularly critical concern in the pediatric population due to the potential long-term risks associated with radiation exposure.

**Fig. 1:**
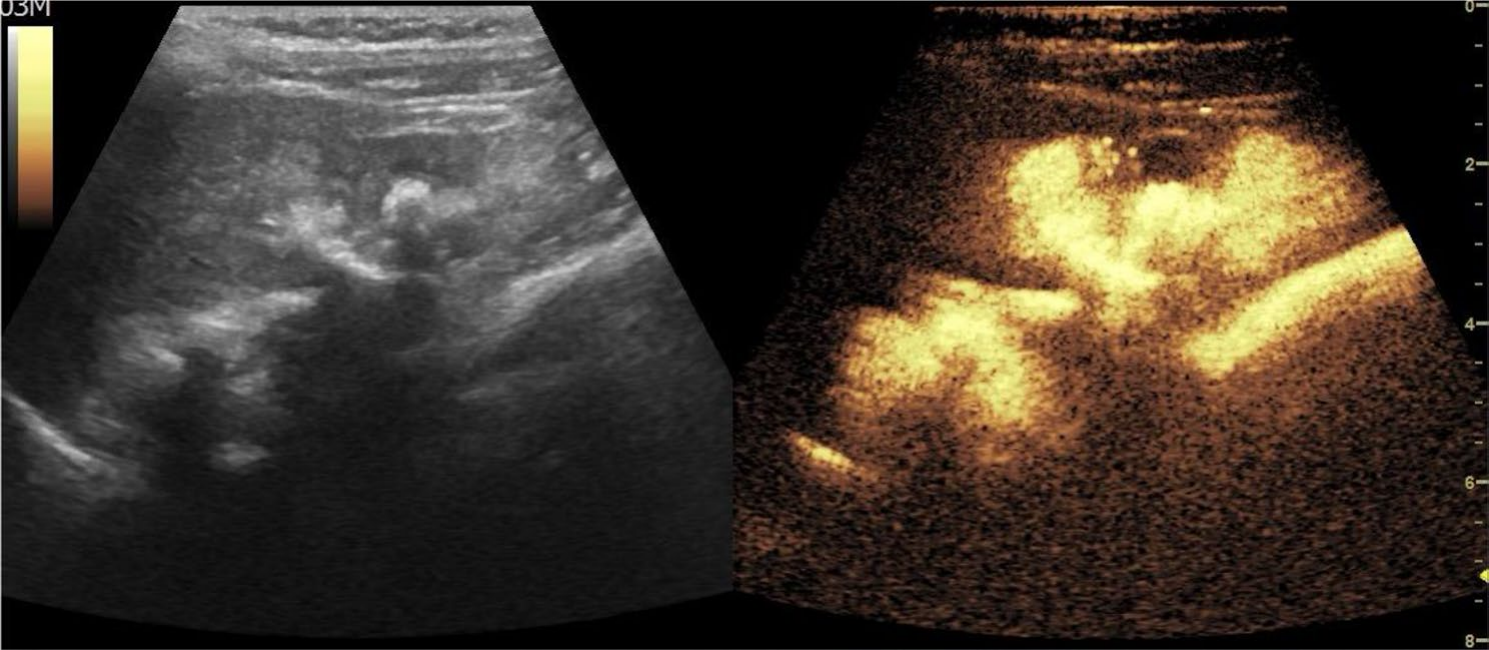
ceVUS examination showing grade 5 VUR in a 5-year-old girl

**Fig. 2:**
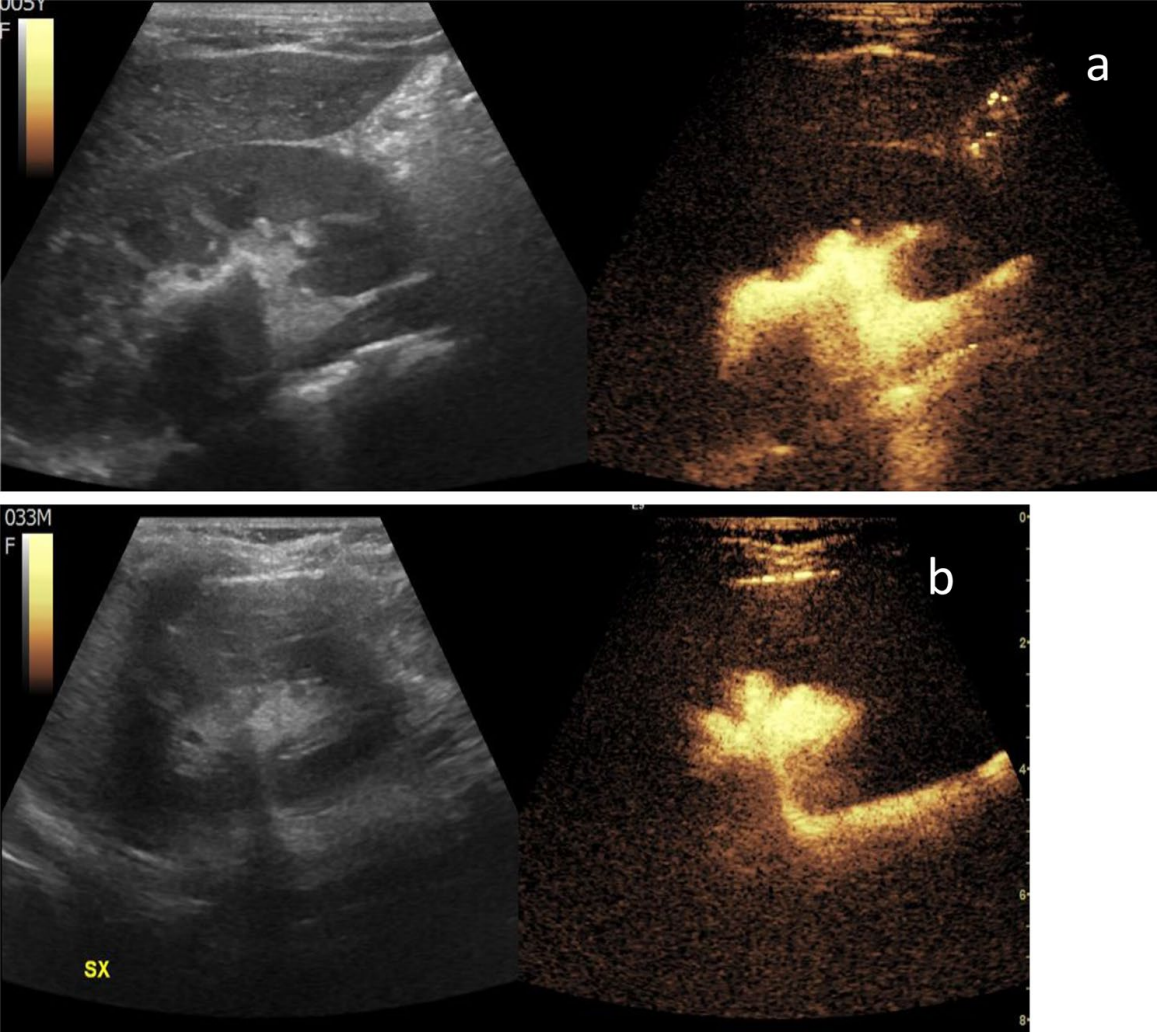
ceVUS examination showing a grade 3 VUR in a 6-year-old girl (**a**) and a grade 4 in a 3-year-old girl (**b**)

Recent advances in imaging technology, particularly contrast-enhanced ultrasound (CEUS), have introduced a promising alternative for monitoring VUR in children, which in this context is referred to as contrast-enhanced voiding urosonography (ceVUS). This method is a non-invasive, radiation-free imaging technique that uses microbubbles as a contrast agent to enhance ultrasound images, allowing for dynamic and real-time evaluation of the urinary tract [2] (Figs. 1 and 2).

Current protocols for the management of pediatric VUR generally involve the use of continuous antibiotic prophylaxis, especially in patients with high-grade VUR (grades III–V), to prevent recurrent UTIs and subsequent kidney damage. However, this strategy often results in prolonged antibiotic treatments, contributing to the growing global issue of antimicrobial resistance [3].

As the timing and frequency of follow-up in patients with VUR remain areas of uncertainty, our study aims to explore the potential of ceVUS in optimizing VUR management, particularly in reducing radiation exposure and the duration of unnecessary antibiotic prophylaxis. By implementing a protocol that utilizes ceVUS instead of traditional VCUG for more frequent follow-ups, we aim to shorten the duration of antibiotic use, thereby mitigating the risk of antimicrobial resistance, while maintaining high diagnostic accuracy and ensuring patient safety.

## Materials and methods

### Study design and population

This observational prospective cohort study was conducted between 2019 and 2024 at our institution, the Santobono Pausilipon Children’s Hospital in Naples, Italy. During the study period, all pediatric patients who presented to our institution with a diagnosis of UTI, hydronephrosis, or a history of VUR were considered for enrollment and included in the study, provided they met the specified inclusion criteria. In particular, the UTIs included in the study involved patients with a single episode associated with ultrasound evidence of urinary tract dilatation, complicated forms such as those with severe sepsis or antibiotic resistance, and recurrent cases.

A total of 1,050 patients were enrolled in this prospective cohort. The study included children aged between 1 and 15 years. The patient recruitment process and follow-up protocol are illustrated in the diagram in Table 1.

**Table 1:**
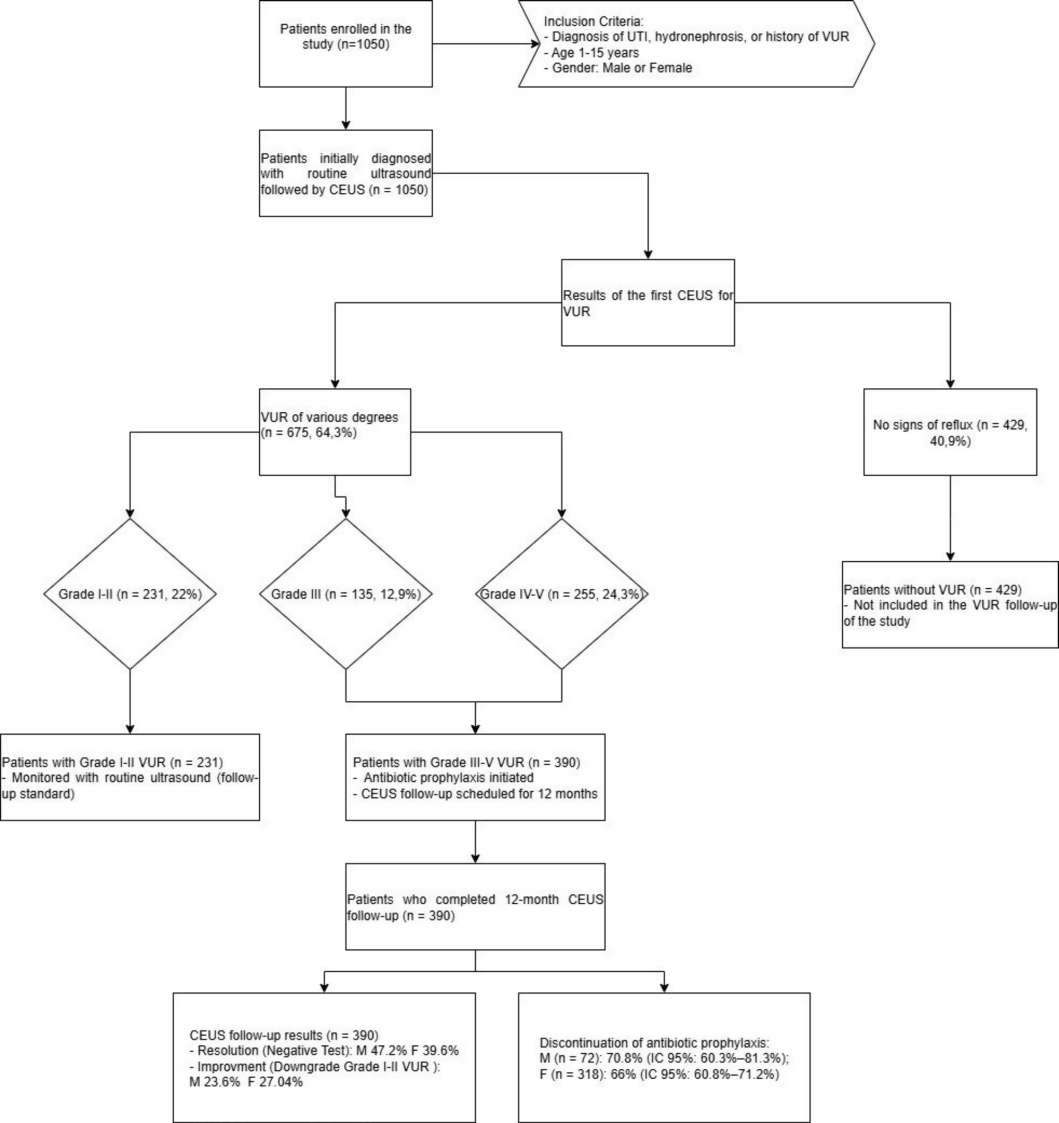
CONSORT-style diagram of patient flow and study protocol

### Stratification criteria

Patients were stratified according to sex and VUR severity. Sex-based stratification included male and female subgroups. VUR severity was classified based on the initial ceVUS diagnosis and categorized into four groups: grade I–II, grade III, grade IV–V, and patients without any evidence of reflux.

### Subgroup analyses

Analyses were conducted based on the aforementioned stratifications. In particular, we evaluated the proportions of patients achieving complete resolution of VUR (i.e., negative test results) or improvement (defined as downgrading to grade I–II VUR) at follow-up, comparing outcomes between male and female patients.

### Statistical analysis

To compare categorical variables such as VUR resolution and improvement between sex-based subgroups, the chi-squared test was used. When the assumptions for the chi-squared test were not met, Fisher’s exact test was applied. A p-value less than 0.05 was considered statistically significant. All statistical analyses were performed using standard software tools, including Microsoft Excel and Jamovi.

### Diagnostic protocols

The diagnosis of VUR was initially confirmed through routine ultrasound, followed by ceVUS to assess the severity of reflux. For patients with grade III, IV, or V VUR, ceVUS was performed 12 months after the initial diagnosis, while those with lower grades were monitored according to the standard protocol with routine ultrasound, typically scheduled one year after the diagnosis.

### Imaging equipment and procedure

Ultrasound and ceVUS exams were performed by experienced pediatric radiologists using convex probes. To improve diagnostic accuracy, the filling and emptying cycles of the bladder were assessed.

The examination had an average duration of about 15–30 min, with images acquired and digitally stored sequentially.

For the ultrasound study of VUR, a second-generation ultrasound contrast agent (SonoVue, Bracco, Milan, Italy) was used. The contrast was pre-mixed by diluting 1 ml of contrast agent into a 100 ml saline bag. Through a bladder catheter, a volume corresponding to the expected bladder volume based on age and weight was introduced.

All patients undergoing ceVUS received antibiotic prophylaxis the day before the examination or were already on prophylactic treatment following a UTI episode.

The examination was performed by a team consisting of a radiologist, a nephrologist, and a nurse, who were respectively responsible for administering the contrast agent, immobilizing the patient, and controlling the urinary catheter.

No sedation was required. Parents provided written informed consent following the regulatory framework of our institution and the country.

## Results

A total of 1050 patients (247 males and 803 females) with a diagnosis of UTI, hydronephrosis, or a history of VUR were enrolled in the study and underwent ceVUS to diagnose the potential presence of vesicoureteral reflux (VUR), as illustrated in the Patient Inclusion and Classification Diagram (Table 2).

**Table 2:**
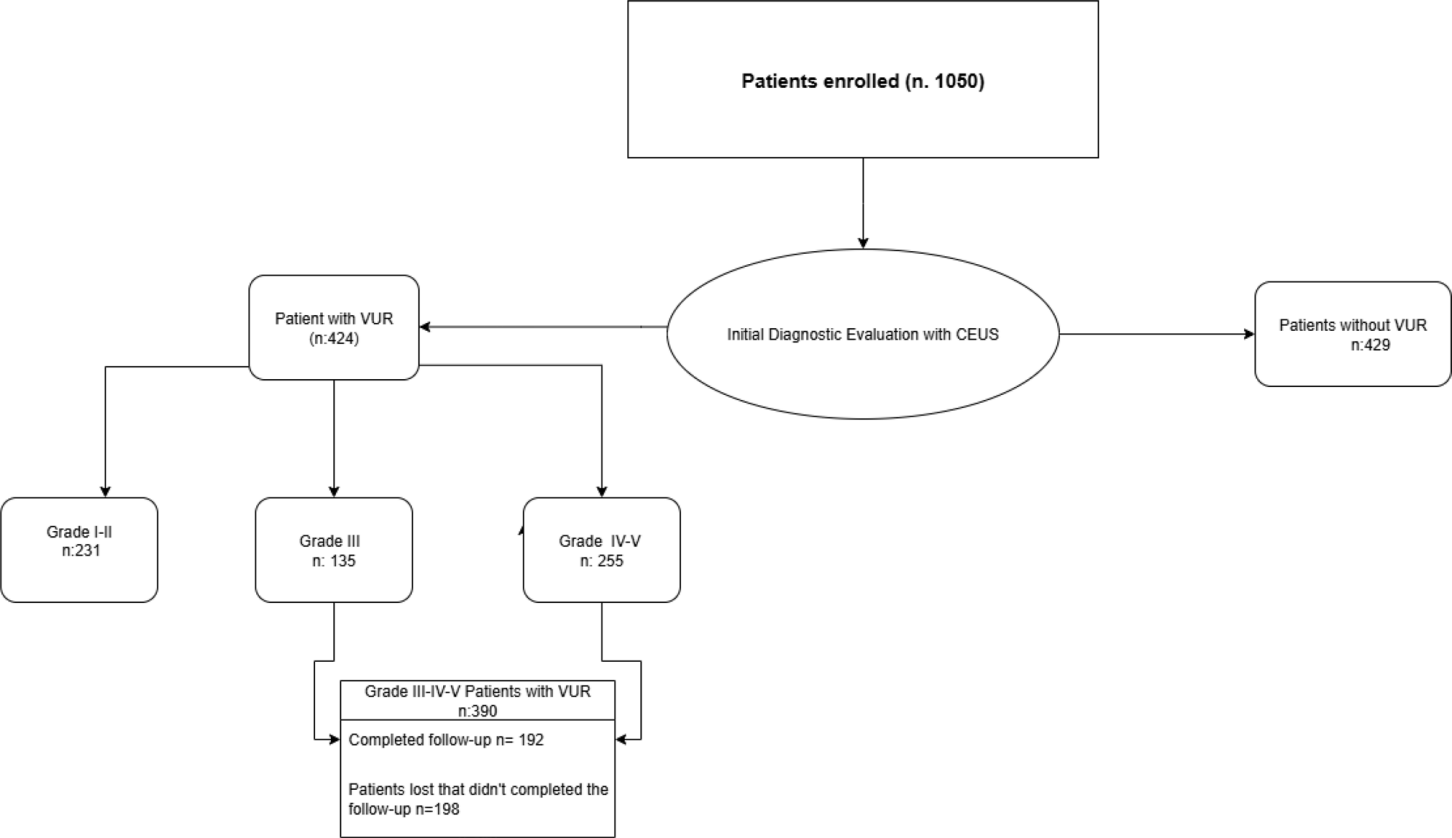
CONSORT-style diagram on patient inclusion

At the first ceVUS, 675 patients (64.3%) presented VUR of varying degrees, distributed as follows: 231 patients (22%) with grade I-II VUR, 135 patients (12.9%) with grade III, and 255 patients (24.3%) with grade IV-V. The remaining 429 patients (40.9%) showed no signs of reflux.

Then, patients with grade III-V VUR (390 patients) were placed on antibiotic prophylaxis and a follow-up CEUS was scheduled for 12 months. Of the 390 patients initially identified with grade III–V VUR, a total of 192 patients (13 males and 60 females with grade IV–V VUR; 8 males and 46 females with grade III VUR) underwent 12-month follow-up with CEUS.

At follow-up, complete resolution of VUR (negative test results) was observed in 47.2% of male patients and 39.6% of female patients, while improvement (downgrade to grade I–II VUR) was recorded in 23.6% of males and 27.04% of females. Data regarding VUR resolution or downgrading in this subgroup are presented in Table 3.

**Table 3:**
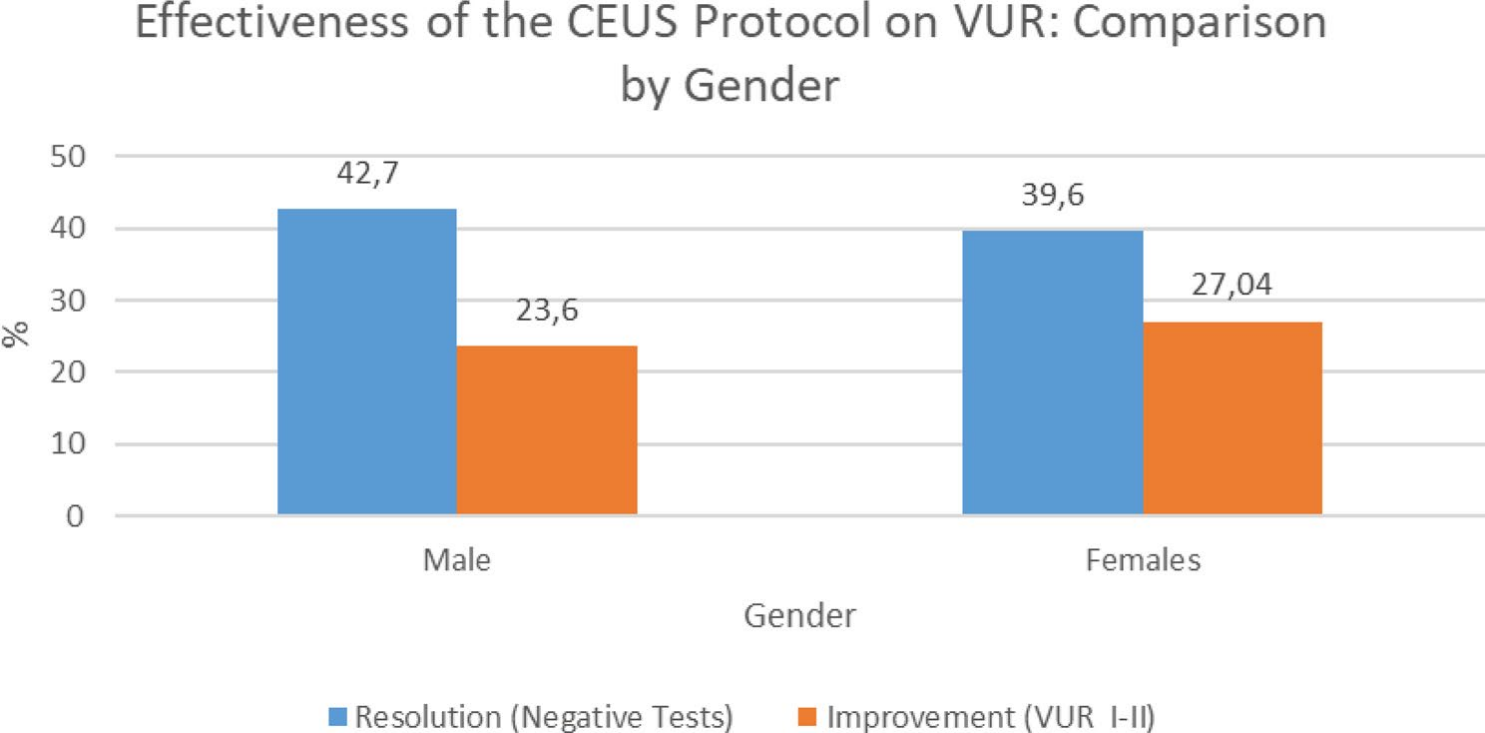
Gender-based comparison of outcomes following the CEUS-based follow-up protocol for vesicoureteral reflux (VUR). The chart illustrates the percentage of male and female patients who showed complete resolution of VUR (negative tests) or improvement (downgrade to VUR grade I–II) at follow-up

Regarding the discontinuation of antibiotic prophylaxis, the mean proportion in males (*n* = 72) was 70.8%, with a 95% confidence interval ranging from 60.3% to 81.3%. In females (*n* = 318), the mean proportion was 66%, with a 95% confidence interval estimated between 60.8% and 71.2% (Table 4), indicating a consistent effect in the studied population.

**Table 4:**
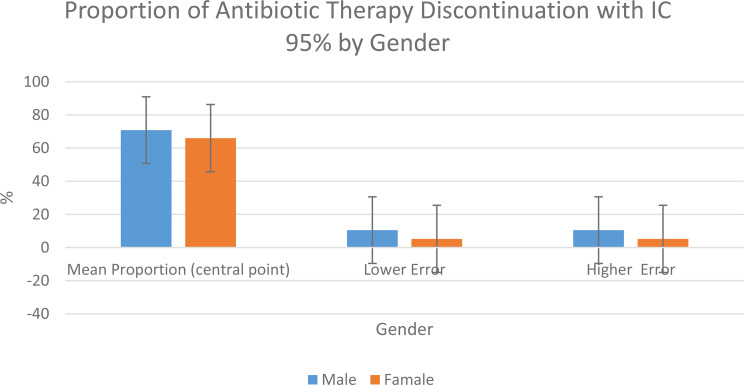
Proportion of antibiotic prophylaxis discontinuation by gender, with 95% confidence intervals. The central bars represent the mean proportion of therapy discontinuation in males (70.8%) and females (66%). Error bars indicate the lower and upper bounds of the 95% confidence intervals for males and females

## Discussion

Vesicoureteral reflux is a common pediatric urological condition that requires careful monitoring due to its potential complications, including kidney damage and recurrent urinary tract infections. However, there is currently no universal consensus on precise guidelines regarding the timing and methods of follow-up for patients with VUR, particularly for those receiving antibiotic prophylaxis.

The most recent American guidelines, updated in 2017, recommend a follow-up ultrasound every 12 months to monitor kidney growth and assess renal parenchyma, with voiding cystourethrography performed after 12–24 months [4]. In patients with additional risk factors, such as high-grade reflux or recurrent UTIs, spontaneous resolution of VUR is less likely, and consequently, a more prolonged follow-up is recommended. The guidelines do not provide a strict protocol on the frequency with which follow-up imaging should be performed within this interval, continuing to consider VCUG as the gold standard for monitoring VUR. This leaves some room for flexibility but also introduces a degree of uncertainty in clinical practice, relying on the clinician’s judgment and the availability of diagnostic resources.

In contrast, the European guidelines, updated in 2024 [5], have confirmed the follow-up interval of 12–24 months, further emphasizing the use of VCUG as the gold standard for the diagnosis and monitoring of VUR. Although these guidelines acknowledge the importance of reducing radiation exposure, they continue to prioritize VCUG in the management of high-grade VUR, especially in cases where spontaneous resolution is unlikely. This represents a challenge both in terms of radiation exposure in pediatric patients and the need for safer and more efficient monitoring methods.

Our study offers new perspectives on how follow-up protocols can be improved.

By using CeVUS, a radiation-free imaging technique that has been considered an effective alternative to VCUG in the latest ESPR recommendations [6] as well (figure no.1 and 2), we have demonstrated the possibility of implementing a more frequent follow-up program, advancing it to 12 months after diagnosis and the start of prophylactic therapy, without the risks associated with ionizing radiation.

This approach resulted in complete resolution of VUR in 47.2% of males and 39.6% of females, and in partial improvement (downgrade to grade I–II) in 23.6% of males and 27.04% of females. These findings suggest that ceVUS follow-up enables earlier identification of patients eligible for discontinuation of antibiotic prophylaxis.

Moreover, the proportion of patients who successfully discontinued antibiotic prophylaxis reached 70.8% in males and 66% in females, with narrow 95% confidence intervals, indicating the reliability of the observed effect. These results further support the clinical utility of ceVUS in reducing the duration of antibiotic exposure in children with VUR, before the 12–24 months typically recommended by traditional protocols.

Since the existing literature reports that ceVUS exhibits high diagnostic accuracy, with a sensitivity of 90% and specificity of 92.8% in the detection of VUR [7] (figure no.3), the more frequent use of CEUS in follow-ups can reduce reliance on VCUG which, although a valuable diagnostic tool, involves radiation exposure and is an invasive and often stressful procedure for pediatric patients. To minimize gonadal exposure to ionizing radiation, VCUG is performed with intermittent X-ray emission in accordance with the ALARA principle. As a result, low-grade (I–II) or transient vesicoureteral reflux may go undetected. In contrast, ceVUS allows continuous, bilateral evaluation of the bladder, ureters, and kidneys, enhancing the detection of even mild reflux (figure no.4). ceVUS has also proven capable of assessing the presence of intraparenchymal flow (figure no.5). The use of ceVUS aligns with the growing trend in pediatric medicine to minimize unnecessary radiation exposure, particularly in children who are more vulnerable to the long-term effects of ionizing radiation.

**Fig. 3:**
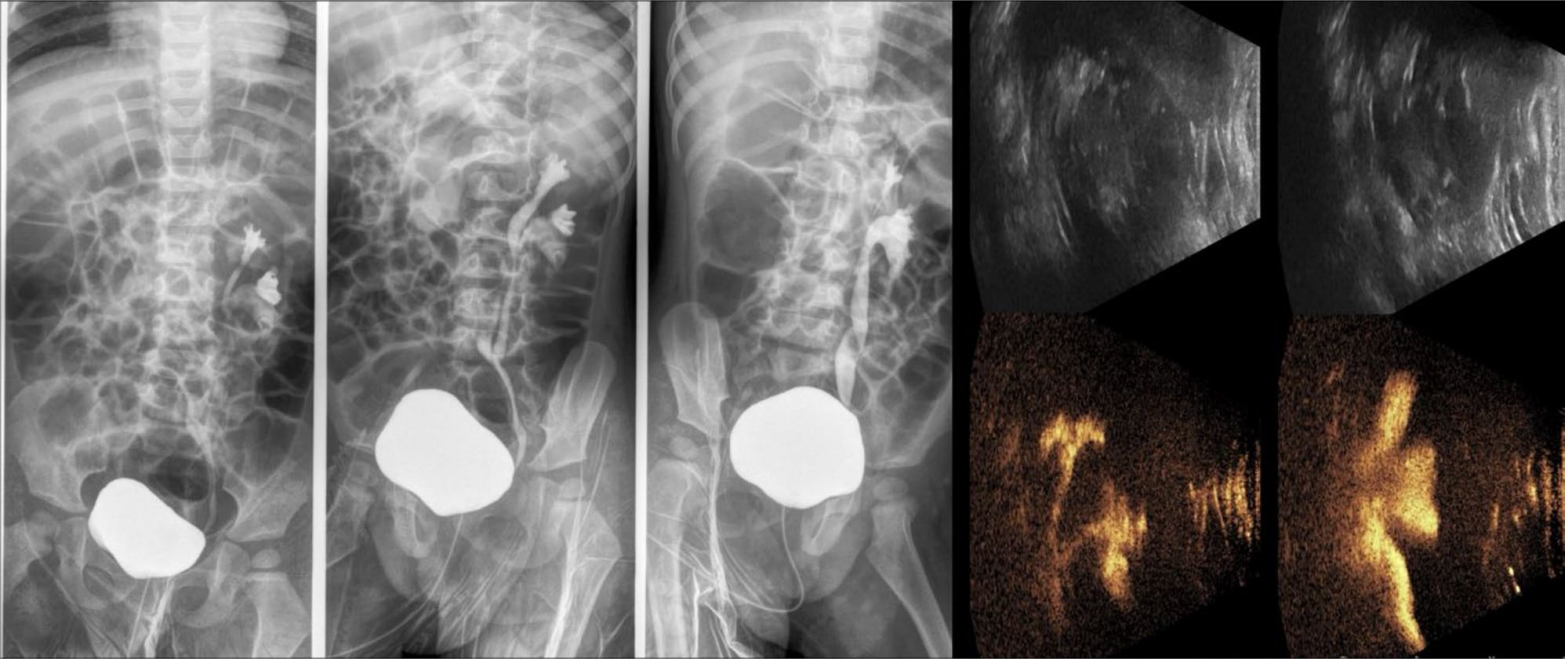
VUR in a 2-year-old girl with repeated urinary infections. VCUG shows findings that correlate well with those depicted at ceVUS

**Fig. 4:**
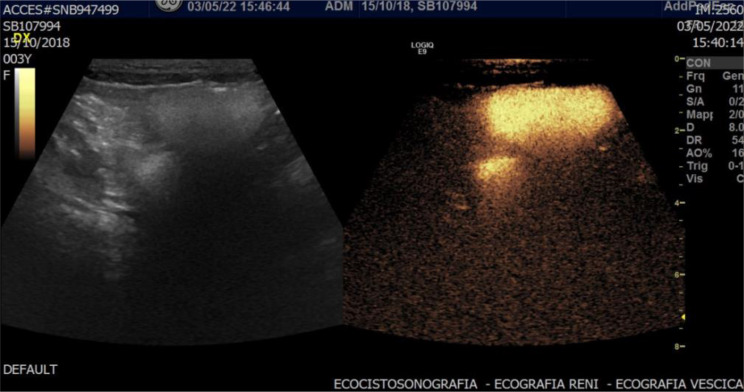
CeVUS demonstrating VUR grade I in a 2-year-old boy

**Fig. 5:**
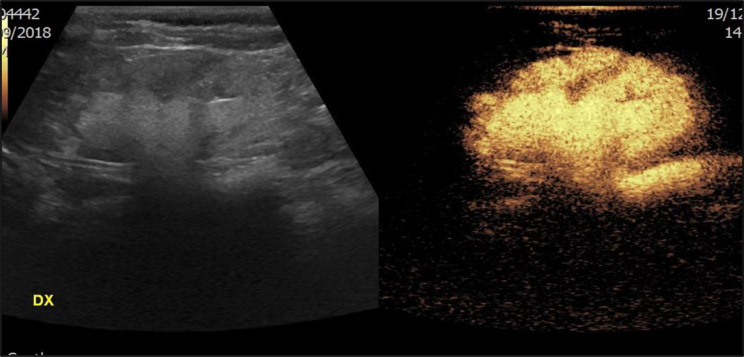
CeVUS demonstrating VUR grade V in a 3-year-old girl with intraparenchymal reflux

Although the results of our study are promising, it is important to emphasize that the timing and optimal method for VUR follow-up will continue to evolve as new data become available. Future studies should further investigate the long-term outcomes of patients managed with ceVUS as part of their follow-up protocol, particularly concerning renal function and VUR resolution.

Overall, our study supports the idea that a more frequent follow-up approach based on radiation-free imaging, such as ceVUS, is both feasible and beneficial in the management of VUR in pediatric patients.

In addition to its diagnostic advantages, this method was associated with earlier and more frequent discontinuation of antibiotic prophylaxis, which has the potential to significantly decrease the risk of antimicrobial resistance while improving patient safety and comfort.

## Conclusions

This study supports the integration of ceVUS into the diagnostic and therapeutic management of pediatric VUR. By reducing radiation exposure and enabling earlier discontinuation of unnecessary antibiotic use, this imaging technique enhances patient safety while contributing to the fight against antibiotic resistance. The adoption of ceVUS as a first-line diagnostic and follow-up tool would therefore represent a significant advancement in optimizing the management of pediatric VUR.

However, despite its promise, ceVUS is not yet universally available, and current European guidelines continue to prioritize VCUG, especially in cases of high-grade VUR. It is important to acknowledge that this study is based on a single-center experience, and multicenter validation is essential before making definitive changes to existing international guidelines.

## Data Availability

All data generated or analysed during this study are included in this published article.

## Abbreviations

VUR: Vesico-ureteral reflux
UTIs: Urinary tract infections
VCGU: Voiding cistoureterography
CEUS: Contrast-enhanced ultrasound
ceVUS: Contrast-enhanced voiding urosonography
ALARA: As low as reasonably achievable
